# Fine motor skills in a population of children in remote Australia with high levels of prenatal alcohol exposure and Fetal Alcohol Spectrum Disorder

**DOI:** 10.1186/s12887-017-0945-2

**Published:** 2017-11-21

**Authors:** Robyn Doney, Barbara R. Lucas, Rochelle E. Watkins, Tracey W. Tsang, Kay Sauer, Peter Howat, Jane Latimer, James P. Fitzpatrick, June Oscar, Maureen Carter, Elizabeth J. Elliott

**Affiliations:** 1 School of Public Health, Curtin University, GPO Box U1987, Perth, WA 6845 Australia; 2 Discipline of Paediatrics and Child Health, Sydney Medical School, The University of Sydney, Sydney, Australia; 3 The George Institute for Global Health, Sydney Medical School, The University of Sydney, Sydney, Australia; 4 Poche Centre for Indigenous Health, Sydney Medical School, The University of Sydney, Sydney, Australia; 5 Physiotherapy Department, Royal North Shore Hospital, Sydney, Australia; 6 Telethon Kids Institute, University of Western Australia, Perth, Australia; 7 Centre for Behavioural Research in Cancer Control, Curtin University, Perth, Australia; 8 Marninwarntikura Women’s Resource Centre, Fitzroy Crossing, Australia; 9 University of Notre Dame, Broome, Australia; 10 Nindilingarri Cultural Health Services, Fitzroy Crossing, Australia; 11 The Sydney Children’s Hospitals Network (Westmead), Sydney, Australia

**Keywords:** Fetal Alcohol Spectrum Disorder, Psychomotor performance, Motor skills, Indigenous population

## Abstract

**Background:**

Many children in the remote Fitzroy Valley region of Western Australia have prenatal alcohol exposure (PAE). Individuals with PAE can have neurodevelopmental impairments and be diagnosed with one of several types of Fetal Alcohol Spectrum Disorder (FASD). Fine motor skills can be impaired by PAE, but no studies have developed a comprehensive profile of fine motor skills in a population-based cohort of children with FASD. We aimed to develop a comprehensive profile of fine motor skills in a cohort of Western Australian children; determine whether these differed in children with PAE or FASD; and establish the prevalence of impairment.

**Methods:**

Children (*n* = 108, 7 to 9 years) were participants in a population-prevalence study of FASD in Western Australia. Fine motor skills were assessed using the Bruininks-Oseretsky Test of Motor Proficiency, which provided a Fine Motor Composite score, and evaluated Fine Manual Control (Fine Motor Precision; Fine Motor Integration) and Manual Coordination (Manual Dexterity; Upper-Limb Coordination). Descriptive statistics were reported for the overall cohort; and comparisons made between children with and without PAE and/or FASD. The prevalence of severe (≤ 2nd percentile) and moderate (≤16th percentile) impairments was determined.

**Results:**

Overall, Fine Motor Composite scores were ‘average’ (M = 48.6 ± 7.4), as were Manual Coordination (M = 55.7 ± 7.9) and Fine Manual Control scores (M = 42.5 ± 6.2). Children with FASD had significantly lower Fine Motor Composite (M = 45.2 ± 7.7 *p* = 0.046) and Manual Coordination scores (M = 51.8 ± 7.3, *p* = 0.027) than children without PAE (Fine Motor Composite M = 49.8 ± 7.2; Manual Coordination M = 57.0 ± 7.7). Few children had severe impairment, but rates of moderate impairment were very high.

**Conclusions:**

Different types of fine motor skills should be evaluated in children with PAE or FASD. The high prevalence of fine motor impairment in our cohort, even in children without PAE, highlights the need for therapeutic intervention for many children in remote communities.

## Background

Local Aboriginal leaders in the remote Fitzroy Valley region of Western Australia introduced alcohol restrictions in 2007 because they were concerned about the social and health effects of chronic alcohol misuse. These concerns included the potential harm caused by alcohol consumption during pregnancy, which can cause Fetal Alcohol Spectrum Disorder (FASD). In 2009 local leaders initiated ‘The Lililwan Project’ (‘Lililwan’ is Kimberley Kriol for ‘all the little ones’) to determine the prevalence of FASD [1]. Diagnoses on the FASD spectrum include Fetal Alcohol Syndrome (FAS) and partial Fetal Alcohol Syndrome (pFAS), both with characteristic facial anomalies and impaired growth; and Alcohol-Related Neurodevelopmental Disorder (ARND) or Neurodevelopmental Disorder – Prenatal/Alcohol Exposed (ND-PAE/ND-AE) with neurodevelopmental impairment in the absence of physical features [2, 3].

PAE can affect the development and function of the corpus callosum [4], cerebellum [5], basal ganglia [6], and motor cortex [7], and children with FASD may have skeletal malformations [8], abnormal muscle development [9], tremor [10], and impaired nerve conductivity [11]. All these factors may impair fine motor performance. Fine motor skills include basic skills such as grip strength, and more complex skills including visual (or fine) motor integration, manual dexterity, and upper-limb coordination. These skills underpin many self-care, academic, and recreational activities, including handwriting, dressing, and ball sports. Fine motor skills are particularly important in primary school aged children, who can spend more than half of their day completing tasks which require fine motor skills [12]. Handwriting quality can be affected by poor fine motor skills, and students with poor handwriting often receive poorer grades [13]. Teacher reports indicate that 20.6% of first year students at Fitzroy Fitzroy Crossing are below the Australian population 10th percentile for fine and gross motor skills [14]. Many Australian Aboriginal students perform below-average on the National Assessment Program – Literacy and Numeracy (NAPLAN), which is conducted annually with students in Years 3, 5, 7, and 9 [15].

Few studies of children with PAE or FASD have reported whether they have a motor impairment, and of those that do, many report a motor score that is a combination of fine motor and gross motor skills [16–18], or a score based on subtests of generalised developmental assessment tools [19], such as the Eye and Hand Coordination subscale from the Griffith’s Mental Development Scales [20]. Individuals with FASD can have subtle neurological impairment, and researchers have highlighted the importance of assessing a range of specific areas of function rather than reporting amalgamated scores [18, 19]. Motor scores that are an average of fine and gross motor skills provide little insight into deficits, which is essential for understanding the child’s neurological profile and developing appropriate therapy goals.

Several studies have assessed a range of fine motor skills in children with PAE or FASD [21–24], but each has used varying assessment tools and none report data from an entire population age-cohort. Motor skills in children with PAE or FASD are summarised in three systematic reviews. In one review, ‘visual and motor’ skills were not associated with mild, moderate, or binge PAE, however, none of the included studies assessed children older than 5 years [25]. Another review found an association between motor impairment and levels of PAE, but did not differentiate between fine and gross motor skills [26]. We reviewed fine motor skills in primary school aged children with PAE or FASD [27], and found that complex fine motor skills, such as visual-motor integration, were more likely to be impaired than basic skills, such as grip strength. We identified a range of assessment tools used to assess fine motor skills in children with PAE or FASD, but few that comprehensively assessed a range of different skills.

### Study hypotheses

Fine motor proficiency and prevalence of impairment amongst children in the remote Fitzroy Valley, Western Australia were evaluated. We hypothesised that rates of fine motor impairment would be high due suspected high rates of neurodevelopmental and socioeconomic risk factors, including PAE. We also hypothesised that children with PAE, particularly those with FASD, would have the most impairment due to the teratogenic effect of alcohol on the central and peripheral nervous systems involved in performance of fine motor skills.

### Study aims

1. Assess and evaluate fine manual control (fine motor precision and fine motor integration) and manual coordination (manual dexterity and upper-limb coordination) in a cohort of children in the Fitzroy Valley.

2. Compare fine motor skills of children (i) without PAE; (ii) with PAE but not FASD; and (iii) with FASD.

3. Determine the prevalence of moderate (≤ 16th percentile) and significant (≤ 2nd percentile) fine motor impairments in the cohort.

## Methods

### Setting

We evaluated fine motor data from the Lililwan Project, a population-based study of FASD prevalence in the Fitzroy Valley in the West Kimberley region of northern Western Australia. The Fitzroy Valley has a population of 4500 people living in communities across a 200 km radius, 80% of whom identify as being Australian Aboriginal [28].

### Procedures

All children born in 2002 or 2003 and living in the Fitzroy Valley during 2010 and 2011 were eligible for inclusion. In Stage 1 of the study parents and carers of 127 children (95% participation) provided information about prenatal and childhood exposures, including PAE, antenatal drug exposures, nutrition, living conditions, and exposure to early life trauma [29]. The Alcohol Use Disorders Identification Test – Consumption (AUDIT-C) was used to classify PAE as ‘low’, ‘risky’, or ‘high risk’ [30].

In Stage 2, 108 of the children completed comprehensive neurodevelopmental assessments by qualified paediatricians and allied health practitioners. Attrition occurred because families moved out of the Fitzroy Valley (*n* = 15); we were unable to locate families or children (*n* = 3); or clinical assessment was declined (*n* = 1).

Assessors were blinded to alcohol and other pre and postnatal exposures. Adapted Canadian FASD Diagnostic Guidelines were used to assign FASD diagnoses, including FAS, pFAS, and ND-AE. To be diagnosed with one of the FASD diagnoses, a child was required to have ‘significant’ impairment (defined as ≥2 *SD* below the mean, or clinically significant variability between subtests on standardised assessments) in a minimum of 3 of 10 neurodevelopmental domains. The diagnoses of pFAS or FAS additionally required evidence of characteristic facial features or growth impairment. A study protocol detailing assessment tools and diagnostic criteria has been published [1]. Children were referred to local health services for medical or therapeutic treatment if required. Families whose child had a FASD diagnosis were referred to a Social Worker and an Indigenous Support Worker with extensive experience working with families affected by FASD. Fine motor skills were assessed in a one hour session by the primary author (RD), an Occupational Therapist with experience working with children in the Fitzroy Valley. Overall motor proficiency and gross motor skills were assessed by a Paediatric Physiotherapist (BRL), and have been reported [31, 32].

### Instrumentation

#### The Bruininks-Oseretsky test of motor proficiency (second edition)

**Fig. 1:**
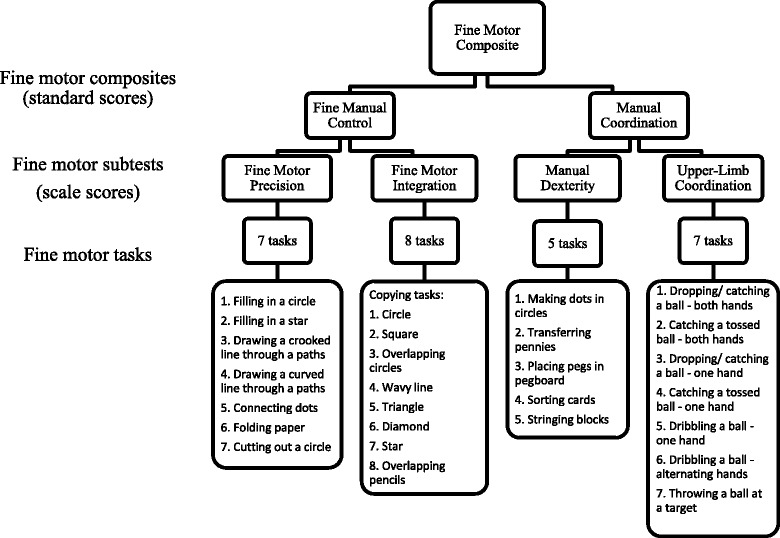
BOT-2 Fine motor composites, subtests, and tasks

The Bruininks-Oseretsky Test of Motor Proficiency (BOT-2) is a standardised, norm-referenced tool suitable for motor assessment in children and young adults aged 4–21 years [33]. Complete (53 tasks) and short versions (14 tasks) are available. The complete version of the BOT-2 was chosen for use in our study because it evaluates a diverse range of fine motor skills; is frequently used in Australia [34] and international FASD diagnostic clinics [35]; and is recommended in the Canadian FASD Diagnostic Guidelines [3]. The BOT-2 provides a Fine Motor Composite score, which is an overall measure of fine motor proficiency. The Fine Motor Composite score is derived from the Fine Manual Control and Manual Coordination composite scores, which in turn are derived from Fine Motor Precision (which assesses precise hand and finger control through paper and pencil tasks, folding paper, and scissor skills), Fine Motor Integration (which assesses ability to reproduce a series of eight geometric shapes), Manual Dexterity (which assess reaching, grasping, and bimanual control through timed tasks such as stringing blocks and placing pegs in a pegboard), and Upper-Limb Coordination (which assesses coordinated arm and hand movement in terms of catching, throwing, and dribbling a tennis ball) subtest scores (Fig. 1). Composites are reported as standardised scores (mean (M) = 50.0, standard deviation (*SD*) = 10.0), and subtest scores are reported as scale scores (M = 15.0, *SD* = 5.0). Descriptive categories are defined as ‘well-above average’(standard score ≥ 70; scale score ≥ 25; ≥ 98th percentile); ‘above average’ (standard score 60 to 69; scale score 20 to 24; 84th to 97th percentile); ‘average’(standard score 41 to 59; scale score 11 to 19; 18th to 83rd percentile); ‘below average’(standard score 31 to 40; scale score 6 to 10; 3rd to 17th percentile); and ‘well-below average’ (standard score ≤ 30; scale score ≤ 5; ≤ 2nd percentile) [33].

BOT-2 tasks are designed to be novel for all children, including those from diverse cultural backgrounds, regardless of familiarity with the tasks, and the composites and subtests have well-established internal consistency and test-retest reliability [33]. The BOT-2 Short Form was trialled in a subset of children from the Lililwan project and we found it to have excellent inter-rater reliability (0.88 to 0.92) and fair to good test-retest reliability (0.62 to 0.73) in this population [35]. The BOT-2 is endorsed as a suitable measure of motor skills in FASD diagnostic assessment [3].

### Statistical analysis

Data were scored using the sex-specific norms of the BOT-2 ASSIST scoring software. The Fine Motor Composite score was calculated using the online Q-global™ scoring system. Means and standard deviations were obtained for all BOT-2 fine motor composite standardised scores and subtest scale scores. Fine motor scores were assessed for normality and analysed using a one-way between groups analysis of variance (ANOVA). Children with unconfirmed or unknown PAE (*n* = 5) were excluded from the between-groups analysis. Group differences were analysed using ANOVA between children without PAE (‘No PAE’ group); children with PAE who did not have multiple, significant neurodevelopmental impairments and were therefore not diagnosed with a type of FASD (‘PAE (no FASD)’ group); and children with confirmed PAE plus FASD (‘FASD’ group). Significance was set at *p* < 0.05. Effect sizes (eta^*2*^) were calculated, with 0.01 being deemed a small effect size; 0.06 a medium effect size; and 0.14 a large effect size [36]. Tukey’s Honestly Significant Difference (HSD) test was utilised as a post-hoc test to determine which groups differed. Prevalence of severe (≥ 2 *SD* below the mean; ≤ 2nd percentile) and moderate (≥ 1 *SD* below the mean; ≤ 16th percentile) impairment was reported for each fine motor composite and subtest for the cohort, and also by exposure group. Statistical analysis was completed using IBM SPSS Statistics for Windows, version 21.0 (Armonk, NY: IBM Corp.).

## Results

### Participants

Participants were aged between 7.5 to 9.6 years (M = 8.7 years) at assessment. The majority were of Australian Aboriginal descent (Table 1). Of the children with PAE (*n* = 60, 55.6%), most (95%) were exposed to ‘risky’ or ‘high risk’ levels according to AUDIT-C criteria [37]. Children who participated in Stage 1 only (*n* = 15) were slightly less likely to have PAE (36.8%) than children who participated in both Stage 1 and 2 (55.6%) but were otherwise similar. Children with and without PAE were born at similar weeks of gestation, and the incidence of pre-term births were also similar [37]. The Universal Non-Verbal Intelligence Test [38] formed part of the assessment battery during the Lililwan Project and was used to evaluate cognitive abilities. Full-scale standard scores were similar between groups with and without PAE or FASD (No PAE M = 89.9, *SD* = 8.5; PAE, no FASD M = 89.4, *SD* = 9.1; FASD M = 85.0, *SD* = 12.3; *p* = 0.329).

**Table 1:** Cohort characteristics

	Total Cohort^a^ *N* = 108	No PAE *n* = 43	PAE (no FASD) *n* = 39	FASD *n* = 21
	*n* (%)	*n* (%)	*n* (%)	*n* (%)
Australian Aboriginal	106 (98.1)			
Gender				
Male	57 (52.8)	24 (55.8)	18 (46.2)	13 (61.9)
Handedness				
Right	101 (93.5)	41 (95.3)	38 (97.4)	19 (90.5)
Hearing^b,c^ (*n* = 93)				
Normal	42 (45.2)	16 (37.2)	14 (35.9)	10 (47.6)
Mild loss	38 (40.9)	15 (34.9)	13 (33.3)	7 (33.3)
Moderate loss	13 (14.0)	7 (16.3)	3 (7.7)	3 (14.3)
Missing	15 (13.9)	5 (11.6)	9 (23.1)	1 (4.8)
Prenatal nicotine exposure^d^				
Yes	67 (62.0)	18 (41.9)	32 (82.1)	15 (71.4)
Unknown	7 (6.5)	0 (0)	1 (2.6)	3 (14.3)
Prenatal marijuana exposure^d^				
Yes	13 (12.0)	2 (4.7)	10 (25.6)	1 (4.8)
Unknown	7 (6.5)	0 (0)	1 (2.6)	2 (9.5)
PAE risk levels^e^				
No exposure	43 (100.0)	0 (0)	0 (0)	0 (0)
Low (1–3)	4 (3.7)	0 (0)	4 (10.3)	0 (0)
Risky (4–5)	4 (3.7)	0 (0)	3 (7.7)	1 (4.8)
High risk (≥ 6)	46 (42.6)	0 (0)	29 (74.4)	17 (81.0)
PAE, uncertain risk	6 (5.6)	0 (0)	3 (7.7)	3 (14.3)
Unknown PAE	5 (4.6)	0 (0)	0 (0)	0 (0)

^a^ ‘Total cohort’ includes *n* = 5 children with unknown PAE who are not included in the No PAE, PAE (no FASD), or FASD groups

^b^ Not all children completed audiology testing

^c^ Mild hearing loss 26 – 40 dB; moderate hearing loss 41 – 55 dB

^d^ Some prenatal exposure information not available, either due to the primary carer not knowing, or the birth mother choosing not to disclose this information

^e^ Risk level according to AUDIT-C scoring criteria

Many children lived in overcrowded households (M = 6.1, range 2–16), and many had lived in more than four homes since birth (*n* = 17, 15.8%). Most children (*n* = 89, 82.4%) attended school 4 to 5 days a week, with only one child (who did not have FASD) not attending school at all. Approximately half (53.3%) of the children’s biological mothers had studied beyond secondary education. These socioeconomic factors were similar between children with and without FASD [39].

### Fine motor composites and subtests

**Fig. 2:**
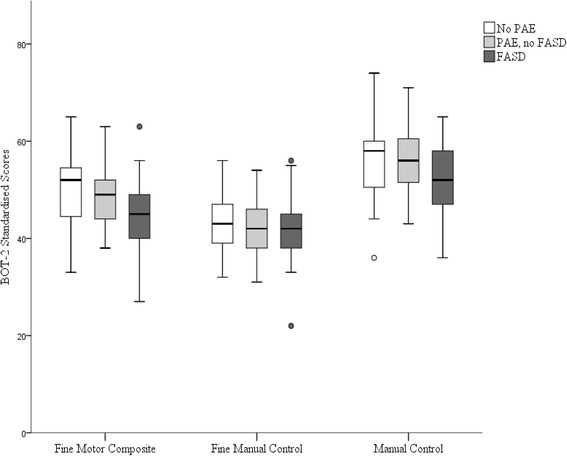
BOT-2 Fine Motor Composite, Fine Manual Control, and Manual Coordination composite scores for children with no PAE; PAE but not FASD; and FASD

For the total cohort, all fine motor composite and subtest scores were in the ‘average’ range (Table 2). Children with FASD had significantly lower Fine Motor Composite scores and Manual Coordination scores than children without PAE (Fine Motor Composite eta^*2*^ = 0.06, Tukey’s HSD *p* = 0.038; Manual Coordination eta^*2*^ = 0.07, Tukey’s HSD *p* = 0.024) (Table 2). There were no other significant differences between groups, but the mean scores of the PAE (no FASD) and FASD groups were consistently lower than in children without PAE in almost all composites and subtests (aside from the Upper-Limb Coordination subtest), and the scores of children with FASD were lower again (Fig. 2).

**Table 2:** BOT-2 Fine motor composite standardised scores and subtest scale scores in children with no PAE; PAE (no FASD); and FASD

	Total Cohort *n* = 108^a^		No PAE *n* = 43		PAE (no FASD) *n* = 39		FASD *n* = 21		ANOVA		
	M (*SD*)	95% CI	M (*SD*)	95% CI	M (*SD*)	95% CI	M (*SD*)	95% CI	df	F	*p*
FINE MOTOR COMPOSITE	48.6 (7.4)	47.2–50.0	49.8 (7.2)	47.6–52.0	48.8 (6.2)	46.8–50.8	45.2 (7.7)	41.7–48.7	2100	3.17	0.046*^d^
Fine Manual Control^b^	42.5 (6.2)	41.3–43.6	43.4 (6.2)	41.4–45.3	41.9 (5.3)	40.2–43.6	41.1 (7.3)	37.8–44.5	2100	1.10	0.336
*Fine Motor Precision* ^c^	12.3 (3.3)	11.7–12.9	12.7 (3.4)	11.7–13.8	11.9 (2.6)	11.0–12.7	11.8 (4.0)	10.0–13.6	2100	0.94	0.393
*Fine Motor Integration* ^c^	11.0 (2.9)	10.5–11.6	11.3 (2.7)	10.4–12.1	11.2 (2.9)	10.3–12.2	10.1 (3.0)	8.8–11.5	2100	1.29	0.279
Manual Coordination^b^	55.7 (7.9)	54.2–57.2	57.0 (7.7)	54.6–59.4	56.2 (7.0)	53.9–58.5	51.8 (7.3)	48.4–55.1	2100	3.74	0.027*^d^
*Manual Dexterity* ^c^	14.9 (3.7)	14.2–15.6	15.4 (3.5)	14.3–16.4	15.1 (3.1)	14.1–16.1	13.2 (4.0)	11.4–15.0	2100	2.97	0.056
*Upper-Limb Coordination* ^c^	19.6 (4.4)	18.7–20.4	19.8 (4.4)	18.5–21.2	20.0 (4.5)	18.5–21.5	18.0 (3.8)	16.3–19.7	2100	1.64	0.200

* *p* < 0.05

^a^ ‘Total Cohort’ includes *n* = 5 children with unknown PAE who are not included in the No PAE, PAE (no FASD), or FASD groups

^b^ BOT-2 norms M = 50, *SD* = 10

^c^ BOT-2 norms M = 15, *SD* = 5. Lower scores represent poorer performance in composites and subtests

^d^ Tukey’s HSD: No PAE > FASD

### Prevalence of fine motor impairment

Prevalence of severe impairment (range 0 to 0.9%) was low in all composites and subtests (Table 3). Prevalence of moderate impairment for the Fine Motor Composite (14.8%) was derived from a high prevalence of moderate impairment in the Fine Manual Control composite (38.9%), and low prevalence in the Manual Coordination composite (1.9%) (Table 3). Only one child with PAE (who had FASD) had severe impairment in any fine motor composite or subtest (Table 3). Prevalence of moderate impairment in the Fine Motor Composite was slightly lower than BOT-2 norms for children without PAE (11.6%) and PAE (no FASD) (7.7%), but much higher in children with FASD (28.6%). Moderate impairment was very high in the Fine Manual Control composite (and its associated subtests) for all exposure groups, but highest in children with FASD (47.6%). Moderate impairment was less than expected in the Manual Coordination composite for all exposure groups (range 0–4.8%), but this composite was an amalgamation of the Manual Dexterity subtest, which had high rates of moderate impairment, particularly for children with FASD (23.8%), and the Upper-Limb Coordination subtest, in which few children had moderate impairment (range 4.7 to 5.1%).

**Table 3:** Prevalence of severe (≥ −2*SD*) and moderate (≥ −1*SD*) fine motor impairment in children with no PAE; PAE (no FASD); and FASD

	Total Cohort *n* = 108^a^	No PAE *n* = 43	PAE (no FASD) *n* = 39	FASD *n* = 21
	*n* (%)	*n* (%)	*n* (%)	*n* (%)
Fine Motor Composite				
- ≥ 2*SD*	1 (0.9)	0 (0)	0 (0	1 (4.8)
- ≥ 1*SD*	16 (14.8)	5 (11.6)	3 (7.7)	6 (28.6)
Fine Manual Control				
-≥ 2*SD*	1 (0.9)	0 (0)	0 (0)	1 (4.8)
-≥ 1*SD*	42 (38.9)*	16 (37.2)*	14 (35.9)*	10 (47.6)**
*Fine Motor Precision*				
- ≥ 2*SD*	1 (0.9)	0 (0)	0 (0)	1 (4.8)
- ≥ 1*SD*	33 (30.6)	12 (27.9)	11 (28.2)	9 (42.9)*
*Fine Motor Integration*				
- ≥ 2*SD*	1 (0.9)	0 (0)	0 (0)	1 (4.8)
- ≥ 1*SD*	48 (44.4)*	17 (39.5)*	15 (38.5)*	13 (61.9)**
Manual Coordination				
- ≥ 2*SD*	0 (0)	0 (0)	0 (0)	0 (0)
- ≥ 1*SD*	2 (1.9)	1 (2.3)	0 (0)	1 (4.8)
*Manual Dexterity*				
- ≥ 2*SD*	1 (0.9)	0 (0)	0 (0)	1 (4.8)
- ≥ 1*SD*	11 (10.2)	5 (11.6)	0 (0)	5 (23.8)
*Upper-Limb Coordination*				
- ≥ 2*SD*	0 (0)	0 (0)	0 (0)	0 (0)
- ≥ 1*SD*	5 (4.6)	2 (4.7)	2 (5.1)	1 (4.8)

- ≥ 2*SD* = ≤ 2nd percentile; − ≥ 1*SD* = ≤ 16th percentile

* = at least twice, and ** = at least three times, the rate of BOT-2 norms

^a^ ‘Total Cohort’ includes *n* = 5 children with unknown PAE who are not included in the No PAE, PAE (no FASD), or FASD group

## Discussion

This is the first study to comprehensively assess fine motor skills in a population-based cohort of predominantly Aboriginal children in Australia. Many children in our study had high levels of PAE and were diagnosed with FASD. The cohort’s mean BOT-2 Fine Motor Composite scores were in the ‘average’ range, an unexpected finding given the high levels of PAE and other neurodevelopmental risk factors in our cohort. However, in keeping with our hypothesis, children with FASD had poorer fine motor skills than children without PAE. Manual coordination skills, including fine motor speed, manual precision, and coordinated arm and hand movement were specific areas of difficulty for children with FASD. Few children had severe impairment (below the 2nd percentile), but rates of moderate impairment (below the 16th percentile) were very high.

Other studies of fine motor impairment in children with PAE or FASD have also reported a mixed profile of strengths and difficulties. A range of assessment tools have been used to evaluate fine motor skills in children with PAE or FASD, including the Visuomotor Precision subtest from the Developmental Neuropsychological Evaluation (NEPSY) [40], the Movement Assessment Battery for Children (M-ABC) [41], and The Beery Buktenica Developmental Test of Visual-Motor Integration (Beery VMI) [42]. Other studies have reported mixed findings for fine motor precision [24, 43] and manual dexterity [44, 45] skills, which weren’t impaired in children with PAE or FASD in our study. Ball skills were also not impaired, which is consistent with other reported findings [44–46]. We found that visual-motor integration (termed ‘fine motor integration’ in the BOT-2) wasn’t impaired, but this contradicts other studies which commonly report visual-motor integration impairment in children with FASD [47–49]. This may be due to the limited number of tasks used to evaluate this skill in the BOT-2 (*n* = 8), compared to the more commonly used Beery VMI (*n* = 30). The Beery VMI formed part of the neurodevelopmental assessment battery in the Lililwan Project, and we reported that the Fine Motor Coordination subtest of the Beery VMI was significantly lower in children with FASD [50].

Only one other study group [17] has published motor outcomes in children with FASD using the BOT. These authors used an earlier version of the BOT (1st edition), which does not include a Fine Motor Composite score. The authors reported that the motor score (an amalgamation of fine and gross motor skills) was not significantly different in children with FASD (M = 49.1) compared to ‘typically developing’ (M = 57.7, *p* = 0.36) children. These non-significant findings may result from areas of stronger skills masking fine motor impairments, in much the same way that children in our cohort with FASD had an ‘average’ Fine Motor Composite score (M = 45.2), which was derived from relatively stronger Manual Coordination (M = 51.8) and weaker Fine Manual Control scores (M = 41.1).

### Implications of prevalence rates

The very low prevalence of severe fine motor impairment in our cohort has implications for FASD diagnosis. The University of Washington 4-digit Diagnostic Code [51] and the Canadian FASD Diagnostic Guidelines [3] each advise that scores 2 *SD* below the mean (≤ 2nd percentile) indicate impairment when diagnosing FASD. In contrast, 1 *SD* below the mean (≤ 16th percentile) indicates impairment according to the Centers for Disease Control (CDC) [2]. Other authors have also proposed a 1 *SD* cut-off for identifying impairment for ND-PAE [52]. Only one child in our cohort (who had FASD) had fine motor scores below the 2nd percentile, which seems conservative given the high levels of PAE and other neurodevelopmental risk factors in our cohort. This issue warrants further consideration and investigation.

### Strengths

This study is the first comprehensive, population-based study of fine motor skills in Aboriginal children in Australia. It is also the first to use a standardised fine motor assessment to develop a comprehensive profile of fine motor skills in children with PAE and/or FASD.

### Limitations

Most children in our study identified as Australian Aboriginal and all were living in remote communities, and so the results should not be generalised. Nevertheless, outcomes may be relevant to other populations with similar demographics. Although the study involved almost two entire age cohorts and had a high participation rate (%), the sample size was too small to statistically control for potentially confounding factors. However, many risk factors, such as early life trauma and low socioeconomic status, were common to almost all children in our study. Many children without PAE also had a moderate level of fine motor impairment, and thus impairments cannot be solely attributed to PAE. However, the high proportion of children in our cohort with “risky” or “high risk” levels of PAE make it likely that PAE contributed, at least in part, to the identified fine motor impairment.

### Recommendations and future directions

This study highlights the importance of comprehensively assessing a range of fine motor skills in children with PAE or suspected FASD. Other researchers have expressed concerns that composite scores may not be sensitive enough to detect subtle neurological impairment in children with FASD [18, 19]. Our findings support these concerns. We recommend that a range of fine motor skills be assessed in children with PAE, and outcomes not be amalgamated with other fine or gross motor scores, because an averaged ‘motor’ score could mask specific difficulties, resulting in inaccurate diagnoses and missed opportunities for therapeutic support.

## Conclusions

Children in our cohort had Fine Motor Composite scores in the ‘average’ range. Upper-limb coordination (ball skills) was a strength, while fine motor integration skills (copying complex shapes) were an area of weakness. Children with FASD had significantly lower Fine Motor Composite and Manual Coordination scores than children without PAE. These outcomes highlight the importance of reporting specific types of fine motor skills, rather than an amalgamated ‘motor’ or even ‘fine motor’ score. The very high levels of impaired fine motor precision and fine motor integration skills highlight the need for therapeutic intervention for many children in the Fitzroy Valley, regardless of PAE, to encourage successful participation in self-care, academic, and recreational activities.

## Abbreviations

ARND: Alcohol-Related Neurodevelopmental Disorder
AUDIT-C: Alcohol Use Disorders Identification Test – Consumption
BOT-2: Bruininks-Oseretsky Test of Motor Proficiency
FAS: Fetal Alcohol Syndrome
FASD: Fetal Alcohol Spectrum Disorder
HSD: Tukey’s Honestly Significant Difference test
NAPLAN: National Assessment Program – Literacy and Numeracy
ND-AE: Neurodevelopmental Disorder – Alcohol Exposed
ND-PAE: Neurodevelopmental Disorder – Prenatal Alcohol Exposed
PAE: Prenatal alcohol exposure
SD: Standard deviation
